# Human epicardial adipose tissue-derived and circulating secreted frizzled-related protein 4 (SFRP4) levels are increased in patients with coronary artery disease

**DOI:** 10.1186/s12933-017-0612-9

**Published:** 2017-10-16

**Authors:** Qingwei Ji, Jianwei Zhang, Yu Du, Enjun Zhu, Zhijian Wang, Bin Que, Huangtai Miao, Shutian Shi, Xiuchuan Qin, Yingxin Zhao, Yujie Zhou, Fangjun Huang, Shaoping Nie

**Affiliations:** 1 Emergency & Critical Care Center, Beijing Anzhen Hospital, Capital Medical University, Beijing, 100029 China; 2 Beijing Institute of Heart, Lung, and Blood Vessel Diseases, Beijing, 100029 China; 3 Department of Cardiology, Beijing Anzhen Hospital, Capital Medical University, Beijing, 100029 China; 4 Beijing Institute of Heart, Lung, and Blood Vessel Diseases, The Key Laboratory of Remodeling-related Cardiovascular Disease, Ministry of Education, Beijing, 100029 China; 5 Department of Cardiac Surgery Center, Beijing Anzhen Hospital, Capital Medical University, Beijing, 100029 China

**Keywords:** Epicardial adipose tissue, SFRP4, Atherosclerosis, Coronary artery disease

## Abstract

**Background:**

Previous studies have demonstrated that secreted frizzled-related protein 4 (SFRP4) is associated with impaired glucose and triglyceride metabolism in patients with stable coronary artery disease. In the present study, we investigated human epicardial adipose tissue (EAT)-derived and circulating SFRP4 levels in patients with coronary artery disease (CAD).

**Methods:**

Plasma samples and adipose biopsies from EAT and subcutaneous adipose tissue (SAT) were collected from patients with CAD (n = 40) and without CAD (non-CAD, n = 30) during elective cardiac surgery. The presence of CAD was identified by coronary angiography. SFRP4 mRNA and protein expression levels in adipose tissue were detected by quantitative real-time PCR and immunohistochemistry, respectively. Plasma SFRP4 concentrations were measured by an enzyme-linked immunosorbent assay (ELISA). Correlation analysis and multivariate linear regression analysis were used to determine the association of SFRP4 expression with atherosclerosis as well as clinical risk factors.

**Results:**

SFRP4 mRNA and protein expression levels were significantly lower in EAT than in paired SAT in patients with and without CAD (all *P* < 0.05). Compared to non-CAD patients, CAD patients had higher SFRP4 expression levels in EAT (both mRNA and protein levels) and in plasma. Multivariate linear regression analysis showed that CAD was an independent predictor of SFRP4 expression levels in EAT (beta = 0.442, 95% CI 0.030–0.814; *P* = 0.036) and in plasma (beta = 0.300, 95% CI 0.056–0.545; *P* = 0.017). SAT-derived SFRP4 mRNA levels were independently associated with fasting insulin levels (beta = 0.382, 95% CI 0.008–0.756; *P* = 0.045). In addition, plasma SFRP4 levels were positively correlated with BMI (r = 0.259, *P* = 0.030), fasting insulin levels (r = 0.306, *P* = 0.010) and homeostasis model assessment of insulin resistance (HOMA-IR) values (r = 0.331, *P* = 0.005).

**Conclusions:**

EAT-derived and circulating SFRP4 expression levels were increased in patients with CAD. EAT SFRP4 mRNA levels and plasma SFRP4 concentrations were independently associated with the presence of CAD.

## Background

Accumulating evidence has established that the pathogenesis of obesity-related metabolic disorders involves a chronic, low-grade inflammatory state. Adipose tissue is a persistent active endocrine organ, secreting numerous pro- and anti-inflammatory adipokines in a paracrine or endocrine pathway, and this secretion pattern is altered in certain pathological conditions involving atherosclerosis [1]. Due to the distinctive location and multifaceted metabolic properties of epicardial adipose tissue (EAT), the association of adipokines derived from EAT and atherosclerosis has been widely investigated [2–4]. Furthermore, a series of adipokines has been shown to accelerate or alleviate the initiation and progression of atherosclerosis, which provides a promising therapeutic target for the treatment of obesity-related diseases including atherosclerosis [5, 6].

Wingless and Int-1 (Wnt) signaling is involved in embryonic development, adipogenesis, carcinogenesis and atherosclerosis [7–10]. The secreted frizzled-related protein family consists of 5 members (SFRP 1-5) in humans and antagonizes Wnt signaling [11]. SFRP4 is an adipokine, and serum SFRP4 levels have been shown to be elevated in patients with different types of diabetes, even a few years before clinical diagnosis of diabetes [12, 13]. Moreover, circulating SFRP4 levels are positively correlated with glucose, insulin, glycated hemoglobin and the homeostasis model assessment of insulin resistance (HOMA-IR) values [14]. Serum SFRP4 levels in patients with stable coronary artery disease (CAD) are also positively correlated with body mass index (BMI), waist circumference and triglycerides, all contributors to metabolic syndrome [15]. In addition, SFRP 4 mRNA levels are increased in visceral adipose tissue from obese individuals and are elevated in human failing hearts due to dilated cardiomyopathy or CAD [16, 17].

Although circulating SFRP4 levels have been widely recognized as a novel biomarker of β-cell dysfunction, insulin resistance and other metabolic disorders, little is known about serum and EAT-derived SFRP4 expression in patients with CAD. EAT is not separated from the myocardium or coronary artery vascular wall by fascia [2]; thus, SFRP4 might have a direct effect on atherosclerosis through a paracrine or vasocrine pathway. To clarify this issue, human SFRP4 expression levels in EAT and plasma were measured in patients with and without CAD who underwent elective cardiac surgery.

## Methods

### Subjects

The study enrolled 70 patients who underwent elective cardiac surgery from January to October 2015. All patients were divided into either a CAD (n = 40) or non-CAD (n = 30) group according to coronary angiography. The CAD group included patients undergoing off-pump coronary artery bypass grafting (CABG) due to left main disease, three-vessel disease or two-vessel disease with a proximal left anterior descending lesion. The non-CAD group included individuals undergoing open-heart surgery for atrial septal defect repair or valvular replacement, with no stenosis found in the coronary artery lumen. The exclusion criteria consisted of age > 75 years, acute myocardial infarction, severe heart failure or cardiogenic shock, active phase of infectious or rheumatic immune disease, liver or renal failure, or pharmacological glucocorticoid or immunosuppressive therapy.

The study complied with the Declaration of Helsinki and was approved by the Ethics Committee of Beijing Anzhen Hospital of Capital Medical University. Written informed consent was obtained from each patient.

### Clinical data collection

Demographic data, body weight, height, medical history and medication use were recorded on admission to the hospital. BMI was calculated as weight (kg) divided by the square of height (m^2^).

### Blood sample measurement

Fasting venous blood samples were obtained from all participants on the morning following admission. Blood samples were collected in sodium heparin vacutainers (Becton–Dickinson) and centrifuged for 15 min at 3000×*g*, and then, plasma samples were stored at − 80 °C. The lipid profiles and levels of fasting glucose, insulin, Glycosylated serum protein and high-sensitivity C-reactive protein (hsCRP) were measured in the central laboratory of Beijing Anzhen Hospital. Insulin sensitivity was evaluated by the HOMA-IR, which was calculated as fasting glucose (mmol/L) × fasting insulin (µU/mL)/22.5.

Plasma SFRP4 levels were measured in duplicate by a commercially available enzyme-linked immunosorbent assay (ELISA) kits (Biomatik, Canada) following the manufacturer’s instructions. The ELISA intra-assay and inter-assay coefficients of variation were both < 5%.

### Adipose tissue acquisition

Paired samples (average 0.3 g each) of EAT and subcutaneous adipose tissue (SAT) were obtained from the proximal right coronary artery and the site of the chest incision from 16 patients with CAD and 13 non-CAD patients, who were randomized selected from CAD group and non-CAD group, respectively. The adipose tissue samples were rinsed with phosphate-buffered saline, followed by division into two portions. One portion was immersed in neutralized formalin for immunohistochemical analysis and the other was frozen in liquid nitrogen for RNA isolation.

### RNA isolation and quantitative real-time PCR

Total RNA was extracted from adipose tissue samples using the Trizol reagent (Invitrogen, USA). The concentration and purity of isolated RNA were evaluated by calculating the ratio of optical density at 260 nm (OD260) and 280 nm (OD 280), and the integrity of RNA was determined by the 18S and 28S ribosomal bands. RNA (2 µg) was used for reverse transcription using the GoScript Reverse Transcription System (Promega, USA). Quantitative real-time PCR analysis was performed with a CFX Real-Time PCR Detection System (Bio-Rad, USA). Each reaction included: 1 µL of cDNA, 0.5 µL of each primer (10 µmol/L), 10 µL of SYBR Premix Ex TaqTM (TAKARA, Japan) and 8 µL of sterile water. The mRNA amplification conditions were 1 min at 95 °C, followed by 44 cycles of 5 s at 95 °C and 30 s at 60 °C, then 0.5 °C increments every 5 s from 55 to 95 °C. All the PCR efficiencies ranged from 90 to 105%.

The primers were designed using Primer Premier 6.0 software (Premier, Canada) with their sites spanning introns. The sequences were as follows SFRP4, forward 5′-GGACCCTGCCAAGTTCAAGA-3′, reverse 5′-ACGGCATACGTGTCGTAGTC-3′; β-actin, forward 5′-AGGTCATCACCATTGGCAAT-3′, reverse 5′-ACTCGTCATACTCCTGCTTG-3′. Threshold cycle values were recorded and relative gene expression was calculated using the formula 2^−ΔΔ CT^.

### Immunohistochemistry

Available EAT and paired SAT samples were randomly selected from the CAD group (n = 8) and the non-CAD group (n = 8). Paraffin-embedded serial biopsy sections were deparaffinized and rehydrated, followed by staining with hematoxylin and eosin. Selected sections were incubated in 3% H_2_O_2_ for 15 min, and then blocked with normal goat serum for 20 min, followed by incubation with the primary antibody (SFRP4, 1:50 dilution, Abcam, USA) at 4 °C overnight in a moisture chamber after removal of excess serum. The slides were incubated with biotinylated secondary antibodies for 20 min, avidin–biotin reagents for 20 min, followed by diaminobenzidine (DAB) and counterstained for 1 min with hematoxylin. High quality images were observed with a light microscope and recorded. Positive staining for SFRP4 was indicated by a brown color. Expression of SFRP4 was quantified by calculating the integrated optical density (IOD) of positively stained tissue using Image-Pro plus software 6.0 (Media Cybernetics, USA). The IOD of each section was calculated from four separate fields viewed at 100 × magnification.

### Statistical analysis

Continuous data are expressed as the mean ± SD or the median (lower quartile, upper quartile), as appropriate. Mean values were compared using Student’s t test, and median values were compared using the Mann–Whitney U test. Categorical variables are expressed as percentages and were analyzed by a Chi square test. The Spearman or Pearson correlation tests were performed to compare SFRP4 levels and clinical variables. The associations between SFRP4 levels and clinical factors, including CAD (present (1)/not present (0)), were determined by univariate analysis and multivariate linear regression analysis. All statistical analyses were performed using SPSS 22.0 software (SPSS, Inc., Chicago, IL, USA). A value of *P* < 0.05 was considered statistically significant.

## Results

### Patient characteristics

The baseline characteristics of the patients are shown in Table 1. Plasma SFRP4 concentrations were significantly higher in patients with CAD than in those without CAD (16.8 ± 3.3 ng/mL vs 14.5 ± 2.3 ng/mL, *P* < 0.001). Compared to non-CAD patients, CAD patients were more likely to have lower total cholesterol and low-density lipoprotein cholesterol levels, partially due to increased patients use of prescription statins in the CAD group. In addition, aspirin and nitrates were also more frequently used by CAD patients. This medication use pattern was also observed in patients with or without CAD who provided adipose tissue. No significant differences in age, sex, BMI, diabetes, hypertension, use of other current medications or of other laboratory examinations were observed.

**Table 1:** Baseline characteristics of patients in the CAD vs non-CAD groups

	Total—CAD (40)	Total—non-CAD (30)	Adipose—CAD^a^ (16)	Adipose—non-CAD^a^ (13)
Age (years)	60.9 ± 6.6	58.2 ± 6.3	59.6 ± 9.0	57.8 ± 6.4
Male (%)	31 (77.5)	17 (56.7)	11 (68.8)	7 (53.8)
BMI (kg/m^2^)	25.9 ± 2.9	24.7 ± 3.0	26.2 ± 2.0	24.5 ± 2.6
LVEF (%)	58.1 ± 9.3	56.2 ± 9.5	62.0 ± 7.9	60.5 ± 5.7
Fasting glucose (mmol/L)	6.2 (5.7, 7.8)	5.4 (5.0, 6.5)	6.6 (5.6, 7.5)	6.2 (5.1, 8.1)
Glycosylated serum protein (%)	15.2 (13.8, 18.3)	15.1 (13.8, 18.5)	17.4 (13.4, 19.4)	14.7 (13.6, 15.7)
Fasting insulin(uU/mL)	36.1 (24.4, 53.3)	24.1 (13.4, 36.2)	35.2 (18.8, 74.4)	21.7 (14.0, 32.1)
HOMA-IR	9.8 (6.7, 15.2)	6.1 (3.4, 10.0)	9.0 (6.4, 26.2)	5.1 (3.6, 9.6)
Triglycerides (mmol/L)	1.5 (1.0, 2.4)	1.5 (1.0, 1.9)	1.9 (1.1, 2.5)	1.2 (0.7, 1.7)
Total cholesterol (mmol/L)	4.0 ± 1.1	4.7 ± 0.9*	4.1 ± 1.1	4.6 ± 0.8
HDL-C (mmol/L)	1.0 (0.8, 1.3)	1.2 (0.9, 1.5)	1.0 (0.8, 1.2)	1.2 (0.8, 1.4)
LDL-C (mmol/L)	2.6 ± 0.9	3.2 ± 0.9*	2.4 ± 0.9	2.8 ± 0.8
hsCRP (mg/L)	1.8 (0.9, 3.1)	1.3 (0.5, 4.0)	2.2 (1.0, 3.0)	1.2 (0.5, 3.0)
SFRP4 (ng/mL)	16.8 ± 3.3	14.5 ± 2.3**	15.7 ± 3.2	13.3 ± 2.4^¶^
Hypertension (%)	19 (47.5)	9 (30.0)	6 (37.5)	5 (38.5)
T_2_DM (%)	14 (35.0)	6 (20.0)	7 (43.8)	2 (15.4)
Smoking (%)	16 (40.0)	7 (23.3)	6 (37.5)	2 (15.4)
Aspirin (%)	34 (85.0)	4 (13.3)**	13 (81.3)	3 (23.1)^§^
Nitrates (%)	38 (95.0)	5 (16.7)**	15 (93.8)	3 (23.1)^§^
ACEI/ARB (%)	14 (35.0)	5 (16.7)	4 (25.0)	2 (15.4)
Statins (%)	23 (57.5)	5 (16.7)**	7 (43.8)	1 (7.7)^¶^
β-Blockers (%)	17 (42.5)	6 (20.0)	8 (50.0)	3 (23.1)
Calcium channel blockers (%)	10 (25.0)	2 (6.7)	4 (25.0)	1 (7.7)
Hypoglycemic agents (%)	13 (32.5)	5 (16.7)	7 (43.8)	2 (15.4)

Data are shown as mean ± SD, median (lower quartile, upper quartile), or number (%)

*CAD* coronary artery disease, *BMI* body mass index, *LVEF* left ventricular ejection fraction, *HOMA-IR* homeostasis model assessment of insulin resistance, *HDL-C* high-density lipoprotein cholesterol, *LDL-C* low-density lipoprotein cholesterol, *hsCRP* high-sensitivity C-reactive protein, *T*_*2*_ *DM* type 2 diabetes, *ACEI/ARB* angiotensin-converting enzyme inhibitor/angiotensin II type 1 receptor blocker

* *P* < 0.05, Total—CAD vs Total—non-CAD

** *P* < 0.01, Total—CAD vs Total—non-CAD

^¶^ *P* < 0.05, Adipose—CAD vs Adipose—non-CAD

^§^ *P* < 0.01, Adipose—CAD vs Adipose—non-CAD

^a^CAD or NACAD patients with adipose tissue available

### Quantitative real-time PCR analysis

**Fig. 1:**
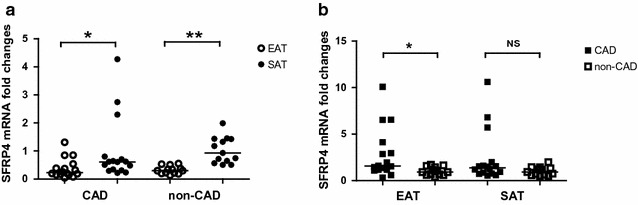
Quantitative real-time PCR analysis for SFRP4 in human adipose tissue. **a** Relative SFRP4 mRNA levels in paired adipose tissue in CAD group (n = 16) and non-CAD group (n = 13). **b** EAT or SAT derived SFRP4 relative mRNA levels in CAD group (n = 16) and non-CAD group (n = 13). *P < 0.05, **P < 0.01. *CAD* coronary artery disease, *EAT* epicardial adipose tissue, *SAT* subcutaneous adipose tissue, *NS* not significant

As shown in Fig. 1a, SFRP4 mRNA levels were significantly lower in EAT than in paired SAT samples in both the CAD group (0.23 vs 0.61, *P* = 0.011) and the non-CAD group (0.19 vs 0.93, *P* = 0.002). Figure 1b shows that EAT SFRP4 mRNA levels were markedly increased in CAD patients compared to non-CAD patients (1.60 vs 0.92, *P* = 0.017), while SAT SFRP4 mRNA levels were not markedly different between the two groups (1.38 vs 0.93, *P* = 0.069).

### Immunohistochemical analysis

**Fig. 2:**
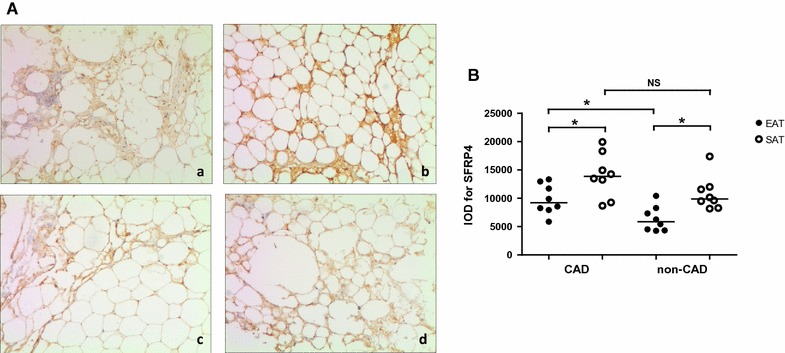
Immunohistochemical analysis for SFRP4 in human adipose tissue. **A** representative slides of adipose tissue from patients in the CAD group (**A**-a, EAT; **A**-b, SAT) and the non-CAD group (**A**-c, EAT; **A**-d, SAT) (magnified × 100). **B** results of quantitative immunohistochemical analysis for SFRP4 in EAT and SAT of the two groups (CAD group, n = 8; non-CAD group, n = 8). *P < 0.05. *CAD* coronary artery disease, *EAT* epicardial adipose tissue, *SAT* subcutaneous adipose tissue, *IOD* integrated optical density, *NS* not significant

Figure 2A shows representative immuno-stained adipose sections from patients in the CAD group (Fig. 2A-a, b) and the non-CAD group (Fig. 2A-c, d), and reveals that SFRP4 protein was expressed in both EAT and SAT, most prominently in the cytoplasm, as well as in the stromal vasculature. As shown in Fig. 2B, SFRP4 protein levels were higher in SAT than in the paired EAT in both the CAD group (13,874.5 vs 9214.5, *P* = 0.039) and the non-CAD group (9883 vs 5844, *P* = 0.016). Furthermore, SFRP4 protein levels in EAT were significantly higher in CAD patients than in non-CAD patients (9214.5 vs 5844, *P* = 0.021), while SFRP4 protein expression in SAT was not different between patients with or without CAD (13,874.5 vs 9883, *P* = 0.105).

### Association of SFRP4 levels with coronary atherosclerosis

Table 2 shows that in the univariate analysis, EAT-derived SFRP4 mRNA levels were positively associated with CAD (beta = 0.430, 95% CI 0.074–0.787). Moreover, this association was found to be independent of age, BMI and fasting glucose in a multivariate linear regression model (beta = 0.442, 95% CI 0.030–0.814; *P* = 0.036). In contrast, as seen in Table 3, SAT-derived SFRP4 mRNA levels were not associated with CAD (*P* = 0.086) but were found to be positively associated with fasting insulin in a multivariate linear regression model adjusted for age, BMI and CAD (beta = 0.382, 95% CI 0.008–0.756; *P* = 0.045). In addition, SAT-derived SFRP4 mRNA levels were positively correlated with HOMA-IR values (r = 0.386, *P* = 0.038).

**Table 2:** Association between EAT SFRP4 mRNA levels and variables using univariate analysis and multivariate linear regression analysis

Variables	Univariate			Multivariate		
	Beta	95% CI	*P* value	Beta	95% CI	*P* value
Age	− 0.028	− 0.423 to 0.366	0.884	− 0.053	− 0.422 to 0.316	0.770
BMI	0.187	− 0.201 to 0.575	0.331	0.158	− 0.270 to 0.585	0.454
Fasting glucose	− 0.145	− 0.536 to 0.246	0.453	− 0.272	− 0.679 to 0.134	0.180
CAD	0.430	0.074 to 0.787	0.020	0.442	0.030 to 0.814	0.036

*EAT* epicardial adipose tissue, *CI* confidence interval, *BMI* body mass index, *CAD* coronary artery disease (presence (1)/not presence (0))

**Table 3:** Association between SAT SFRP4 mRNA levels and variables using univariate analysis and multivariate linear regression analysis

Variables	Univariate			Multivariate		
	Beta	95% CI	*P* value	Beta	95% CI	*P* value
Age	− 0.180	− 0.569 to 0.208	0.350	− 0.234	− 0.587 to 0.120	0.185
BMI	0.251	− 0.131 to 0.633	0.189	0.084	− 0.300 to 0.468	0.656
Fasting insulin	0.452	0.100 to 0.804	0.014	0.382	0.008 to 0.756	0.045
CAD	0.325	− 0.049 to 0.698	0.086	0.214	− 0.171 to 0.600	0.263

*SAT* subcutaneous adipose tissue, *CI* confidence interval, *BMI* body mass index, *CAD* coronary artery disease (presence (1)/not presence (0))

Plasma SFRP4 levels were positively correlated with the BMI (r = 0.259, *P* = 0.030), fasting insulin levels (r = 0.306, *P* = 0.010) and HOMA-IR values (r = 0.331, *P* = 0.005). As shown in Table 4, multivariate linear regression analysis indicated that plasma SFRP4 levels were independently associated with the presence of CAD (beta = 0.300, 95% CI 0.056–0.545; *P* = 0.017) after adjusting for age, BMI, HOMA-IR and triglycerides.

**Table 4:** Association between serum SFRP4 levels and variables using univariate analysis and multivariate linear regression analysis

Variables	Univariate			Multivariate		
	Beta	95% CI	*P* value	Beta	95% CI	*P* value
Age	0.158	− 0.081 to 0.397	0.191	0.076	− 0.154 to 0.306	0.513
BMI	0.224	− 0.012 to 0.460	0.062	0.094	− 0.151 to 0.340	0.446
HOMA-IR	0.275	0.043 to 0.508	0.021	0.171	− 0.075 to 0.417	0.170
Triglycerides	− 0.116	− 0.356 to 0.125	0.341	− 0.017	− 0.251 to 0.217	0.884
CAD	0.373	0.149 to 0.598	0.001	0.300	0.056 to 0.545	0.017

*CI* confidence interval, *BMI* body mass index, *HOMA-IR* homeostasis model assessment of insulin resistance, *CAD* coronary artery disease (presence (1)/not presence (0))

## Discussion

Many studies have indicated that adipokines, such as adiponectin, leptin, chemerin and omentin, serve as biomarkers of CAD [3–6]. The association of adiponectin with CAD has been widely investigated in previous studies [4, 18–23]. We demonstrated that both circulating and EAT adiponectin levels are decreased in CAD patients [4, 18]. Additionally, we found that adiponectin deficiency in perivascular adipose tissue promoted atherosclerosis [19]. Moreover, Wang et al. reported that over-expression of adiponectin significantly inhibited the formation of atherosclerotic plaques in ApoE−/− mice [20], suggesting a protective role of adiponectin in atherosclerotic disease. Furthermore, accumulating clinical evidence has demonstrated that circulating adiponectin levels are not only associated with the onset of CAD but are also independently associated with cardiovascular mortality [18, 20–23]. Recently, we found that the levels of circulating and EAT omentin-1 were decreased in CAD patients compared to non-CAD patients [4]. Furthermore, CAD is an independent predictor of EAT and circulating omentin-1 levels. Taken together, these findings support a close association of EAT-derived adipokines with the onset of CAD.

In the present study, we found that EAT and circulating SFRP4 levels were increased in CAD patients compared to non-CAD patients. However, the expression of SAT SFRP4 was not different in these groups. Interestingly, plasma SFRP4 levels were positively correlated with BMI, fasting insulin levels and HOMA-IR values, which were not revealed in EAT SFRP4. Additionally, CAD was an independent predictor of the increased EAT and plasma SFRP4 levels. The results suggest that SFRP4 is a novel biomarker of CAD and might play a role in the development of CAD.

SFRP4 is a member of the SFRP family that was identified as a heparin-binding polypeptide in conditioned medium from a human embryonic lung fibroblast line in 1997 [24]. Immature SFRP4 is a 36 kDa protein that contains a signal peptide, an N-terminal cysteine-rich domain that is 30–40% identical to a putative Wnt-binding domain of Frizzled, and a hydrophilic carboxyl terminus. Additionally, the circulating form of mature sFRP4 is a 48 kDa protein after posttranslational glycosylation. The SFRP4 gene is also found in monkeys, mice, pigs and toads, but is not detected in fruit flies or yeast. SFRP4 expression displays temporal and spatial characteristics during embryonic development. Additionally, SFRP4 is expressed in a tissue-specific manner in adult humans, with the highest expression in the heart, followed by the kidneys, ovaries, prostate, testis, small intestine and colon, while the placenta, brain tissue and pancreas exhibit low expression levels [24]. However, SFRP4 was not detected in the lung, liver, thyroid or white blood cells.

SFRP4 binds directly to Wnt, modulates both canonical and non-canonical Wnt pathways and is therefore involved in the embryonic developmental pathway and adult pathological processes. For example, many studies have identified a close association of SFRP4 with tumors [25–30]. SFRP4 mRNA is over-expressed in primary serous ovarian tumors but decreased in prostate cancers, endometrial stromal sarcomas, lung squamous cell carcinoma and pancreatic tumors [25–29]. Low SFRP4 expression is associated with an unfavorable prognosis in prostate and ovarian cancer [26, 30]. Additionally, some studies have shown that SFRP4 participates in apoptosis [31, 32], angiogenesis [33, 34], and bone formation [35, 36]. Notably, Matsushima et al. reported that the expression of SFRP4 in the heart was increased in a rat infarction model, while treatment with recombinant SFRP4 reduced fibrosis scar size and improved impaired heart function [37], suggesting a protective role of SFRP4 in myocardial infarction, which is a severe form of CAD.

Recently, accumulating evidence has identified SFRP4 as a novel adipokine [11–17]. Ehrlund et al. found that SFRP4 is secreted from human white adipose tissue (WAT), and SFRP4 expression is up-regulated both in human SAT and visceral WAT in obese compared to lean subjects [17]. These results are consistent with the findings of Garufi [38], who reported that circulating and abdominal SAT SFRP4 levels were significantly increased in obese individuals and abdominal SAT is the main source of circulating SFRP4 in obese subjects. In the present study, we found that EAT SFRP4 expression significantly increased in CAD patients compared to non-CAD patients, although EAT SFRP4 expression was lower than SAT SFRP4 expression both in patients with and without CAD, suggesting that the increase in circulating SFRP4 can be partially accounted for by the high expression of EAT SFRP4 in CAD patients. SFRP4 plays a critical role in the control of adipogenesis. Park et al. found that SFRP4 mRNA was up-regulated gradually during adipogenic differentiation in human adipose tissue-derived mesenchymal stem cells (hAMSCs) [11]. Moreover, blocking SFRP4 inhibition with small interfering RNA inhibited differentiation of hAMSCs into adipocytes and restored β-catenin levels, suggesting that SFRP4 promotes adipocyte differentiation via suppressing the canonical Wnt pathway [11]. Additionally, many studies have demonstrated that SFRP4 is involved in glucose and lipid metabolism and insulin secretion [12, 32]. By analyzing global gene expression in human pancreatic islets, Mahdi et al. found that, accompanied by the expression of inflammatory markers, SFRP4 was up-regulated in patients with type 2 diabetes [12]. SFRP4 treatment resulted in a decrease in insulin secretion and glucose intolerance, while silencing of SFRP4 led to glucose-stimulated insulin release [12]. Furthermore, they found that not only type 2 diabetes patients but also patients who later developed type 2 diabetes had higher serum SFRP4 levels than the controls, although the sample size was small. These clinical results were also demonstrated by later studies [13, 14], which found that SFRP4 levels were significantly increased in impaired glucose tolerance patients and patients with different types of diabetes including type 1 diabetes, type 2 diabetes and latent autoimmune diabetes of the adult (LADA). However, using a diet-induced obesity model, Mastaitis et al. found that SFRP4 deficient mice have normal glucose and insulin levels [39]. These disparate results may be associated with the use of different models. In the present study, we found that SAT SFRP4 mRNA levels were positively correlated with fasting insulin, and circulating SFRP 4 levels were positively correlated with HOMA-IR, although no significant correlation between EAT SFRP4 and fasting insulin was observed. These results may be owed to small samples of the present study. Therefore, the effect of SFRP4 on insulin-related metabolism should be further investigated.

Numerous studies have demonstrated that Wnt pathway-related proteins are highly expressed in atherosclerotic lesions, participate in cholesterol transportation, modulate the inflammatory process and thereby play a pivotal role in atherosclerosis [5, 6, 10]. These findings also indicated that SFRP4 may participate in atherosclerosis through regulation of the Wnt pathway. Recently, circulating SFRP4 levels were detected in 504 patients with stable CAD [15]. The results showed that patients with metabolic syndrome, insulin therapy, diabetes and a history of myocardial infarction or percutaneous intervention had higher SFRP4 levels. Correlation analyses have revealed that elevated SFRP4 levels are positively correlated with HbA1c, fasting insulin, BMI and fasting and postprandial triglyceride levels, and these results were also found in our study. After 48 months of follow-up, the baseline SFRP4 level was not associated with the onset of the primary cardiovascular endpoint, although patients experiencing a stroke/transitory ischemic attack had increased SFRP4 levels. This was the first study focusing on SFRP4 in CAD patients. However, since patients without CAD were not enrolled as controls in that study, it is difficult to conclude that the changes in circulating SFRP4 levels were associated with CAD. Therefore, SFRP4 levels of CAD patients and controls were measured in the present study to determine whether SFRP4 levels increase and are associated with the presence of CAD. Although the controls were not healthy subjects and the sample size was small, the results showed that EAT SFRP4 expression was significantly increased in CAD patients, and CAD is an independent predictor of SFRP4 elevation, indicating that SFRP4 elevation is attributed to the onset of CAD, and participates in the development of CAD via modulation of the Wnt pathway. Furthermore, similar results were obtained regarding plasma SFRP4 levels in both groups.

There are some limitations to our study. First, the sample size is small and should be expanded in the future. Second, circulating SFRP4 levels should be measured in a future study in which patients with different clinical types of CAD, including stable angina, unstable angina and myocardial infarction, as well as healthy controls, are enrolled. Only in this way can the changes in SFRP4 in CAD be clearly distinguished.

In conclusion, our study found for the first time that EAT and plasma SFRP4 levels were increased in patients with CAD. Additionally, both EAT and plasma SFRP4 levels were independently associated with the presence of CAD. Therefore, these results indicated that the novel adipokine SFRP4 is involved in CAD. However, the exact role of SFRP4 in CAD and atherosclerosis remains unknown and should be investigated in prospective studies.

## Abbreviations

SFRP4: secreted frizzled-related protein 4
EAT: epicardial adipose tissue
SAT: subcutaneous adipose tissue
CAD: coronary artery disease
PCR: polymerase chain reaction
ELISA: enzyme-linked immunosorbent assay
Wnt: Wingless and Int-1
HOMA-IR: homeostasis model assessment of insulin resistance
BMI: body mass index
CABG: coronary artery bypass grafting
hsCRP: high-sensitivity C-reactive protein
C_T_: threshold cycle
DAB: diaminobenzidine
IOD: integrated optical density
HDL-C: high-density lipoprotein cholesterol
CI: confidence interval
